# Factors predicting self-report adherence (SRA) behaviours among DS-TB patients under the “Integrated model”: a survey in Southwest China

**DOI:** 10.1186/s12879-022-07208-6

**Published:** 2022-03-01

**Authors:** Rui Zhang, Jie Pu, Jiani Zhou, Qingya Wang, Ting Zhang, Shili Liu, Geng Wang, Yong Chen, Jiaqing Liu, Daiyu Hu, Ying Li

**Affiliations:** 1 Department of Social Medicine and Health Service Management, Army Medical University (Third Military Medical University), Chongqing, China; 2 Department of Districts and Counties, Chongqing Institute of TB Prevention and Treatment, Jiulongpo District, Chongqing, China

**Keywords:** Drug-sensitive tuberculosis, Self-report adherence behaviours, Factors, Southwest China

## Abstract

**Background:**

China is one of 30 countries with a high tuberculosis (TB) burden, and poor adherence to TB treatment is one of the biggest challenges for TB control. We aimed to explore the barriers and facilitators of treatment adherence among drug-sensitive tuberculosis (DS-TB) patients under the “Integrated model” in Western China, to provide evidence-based treatment and control regimens for DS-TB patients to improve adherence behaviours.

**Methods:**

Both qualitative and quantitative research methods were used to explore the factors associated with self-reported adherence (SRA) behaviours. Questionnaire surveys with DS-TB patients and in-depth interviews with leaders from the Centers for Disease Control and Prevention (CDC) and community health sectors (CHCs), healthcare workers (HCWs) from CHCs, and DS-TB patients were conducted.

**Results:**

A total of 459 eligible patients were included in the quantitative survey, and two patients and 13 healthcare providers were included in the in-depth interviews. The percentage of patients who experienced a missed dose, lack of follow-up sputum examination, and interrupted treatment were 19.0%, 11.3%, and 9.2%, respectively. Patients aged 20–39 had a higher risk of missed dose [OR (95% CI): 2.302 (1.001–5.305)] and a lower risk of interrupted treatment [OR (95% CI): 0.278 (0.077–0.982)] than patients more than 60 years. Patients who were of Han ethnicity (OR [95% CI]: 0.524 [0.301–0.912]) received psychological support (OR [95% CI]: 0.379 [0.144–0.998]) from their family and had a lower risk of missed doses. Patients who had drug side effects had a higher risk of interrupted treatment (OR [95% CI]: 2.587 [1.237–5.412]). Patients who possessed higher knowledge had a lower risk of lack of follow-up sputum examination [OR (95% CI): 0.817 (0.673–0.991)]. The results of the qualitative study also reported that patients’ poor TB knowledge was the main reason for their non-SRA behaviours.

**Conclusions:**

Patient-centred strategies should be implemented to improve health literacy and strengthen psychological support. More effective case management should be designed and implemented based on different patient characteristics to improve adherence behaviours in further studies.

## Background

Tuberculosis (TB) is a communicable disease with a long history [1, 2], and one of the top 10 global causes of death and the leading cause of death from a single infectious agent (higher than HIV/AIDS) [1]. According to the World Health Organization (WHO) Global TB Report 2020, 10.0 (8.9–11.0) million people fell ill with TB in 2019, and 3.3% of new cases and 18% of previously treated cases developed multidrug-resistant TB (MDR-TB) or rifampicin (RFP)-resistant TB (RR-TB) [1]. China is one of the 30 high TB burden countries with an estimated 833,000 TB patients in 2019, accounting for 8.4% of global TB patients [1]. TB is life-threatening, curable, and preventable [3]. The WHO estimated the worldwide treatment success rates to be 85% and 57% for drug-sensitive TB (DS-TB) and MDR-TB/RR-TB patients, respectively [1]. In China, the treatment success rates for DS-TB and MDR-TB/RR-TB patients were 94% and 54%, respectively, while the loss to follow-up was the second-highest in the world (29%) [1]. The treatment success rate worldwide for DS-TB and MDR-TB/RR-TB patients was much lower than that estimated by the WHO. One of the main challenges in the treatment of TB is non-adherence to treatment due to the long treatment period, modest tolerability, and complexity of drug regimens [4, 5]. Non-adherence behaviours among TB patients include missed dose, lack of follow-up, interrupted treatment, etc. [6, 7]. Non-adherence to TB treatment increases the risk of morbidity, mortality, and drug resistance [8].

In response of non-adherence to treatment for TB patients, the WHO proposed direct observation of treatment and short course (DOTs), which needs to be monitored on a regular basis to maintain treatment adherence for six to eight months for DS-TB patients and 18–24 months for MD-TB/ RR-TB [9, 10]. DOTs have been implemented globally and have improved adherence to TB treatment [11–13]. However, both patients and healthcare providers are facing challenges in DOT implementation [7, 14–18]. In China, DOTs were implemented in 1991 and achieved 100% coverage in 2005 [19]. However, previous studies have reported that DOTs have not been implemented effectively for all TB patients under the “Centers for Disease Control and Prevention (CDC) model” [20, 21]. Consequently, in 2011, during the 12th National TB Five-Year-Plan program, there is a novel “integrated model” that has been proposed by the administration [22]. In its framework, every subject has clear responsibility boundaries. Primary healthcare centers are in charge of case management and patient referral. Designated hospitals are primarily in charge of TB patients’ diagnosis and treatment. The CDC is in charge of TB control, concrete duties include supervision, planning, and health education [22].

There is an average of 695 per 100,000 infection rate of TB in the western part of China and an average of 463 per 100,000 and 291 per 100,000 infection rate in the central and eastern part of China that shows the western part of China suffers the higher TB prevalence than in the eastern and central part of China [4]. Southwest China is an underdeveloped area in China, whose per capita net income is much lower than that of eastern and central China [23]. Chongqing and Guizhou are the high TB burden municipality /province in Southwest China [24]. According to a study report in 2015, Guizhou and Chongqing ranked third (133.5/100,000) and tenth (75.0/100,000) highest TB incidence rates in China, respectively [24]. TB preventive and control programs faced great challenges in southwest China. Our previous study evaluated the TB case management status in Guizhou and Chongqing under the “integrated model”, and we found that the standard TB case management are far below the requirements and need improvement, and are also with low adherence to treatment [21, 25]. It is crucial to identify the barriers and facilitators of adherence to treatment to improve treatment outcomes for patients with TB [26]. A systematic review concluded that multiple factors affected non-adherence behaviours, including patient-centred, social, economic, health system, and therapy factors [7].

This study aimed to evaluate the self-reported adherence (SRA) behaviours and the association between the above factors and SRA behaviours under the “integrated model’ among DS-TB patients in Southwest China, and to explore the reasons for poor adherence behaviours from the perspectives of both healthcare demanders (patients) and healthcare providers, to further provide evidence-based treatment and control regimens for DS-TB patients.

## Methods

A cross-sectional study was conducted in Guizhou and Chongqing using both qualitative and quantitative research methods to explore the factors predicting SRA behaviours among patients with DS-TB.

### Quantitative study

#### Participants

Consecutive sampling was used to recruit patients with DS-TB as participants for the quantitative study. Participants who enrolled in our study met the inclusion and exclusion criteria. The inclusion criteria were as follows: (1) TB patients who were registered in a TB dispensary; (2) patients aged > 15 years; (3) received at least four months of TB treatment and no more than four months after complete treatment. Exclusion criteria: (1) patients with intellectual disability and difficulty in speech and hearing; (2) patients with severe complications (such as heart failure, cerebral infarction, and multiple organ failure) or who underwent hospitalisation; and (3) patients who refused to participate in this survey.

#### Data collection

This questionnaire was designed by our research team, who reviewed the existing literature reports and then consulted related experts before the pilot study. Then, a pilot test was conducted with 100 participants, and the Cronbach’s alpha value of the pilot study was 0.753. Trained investigators executed all questionnaires from our research group, and the completed questionnaires were checked and examined by trained investigators for quality control.

A self-designed structured questionnaire was administered to collect data, including socio-demographic information, knowledge about TB (knowledge indicators including transmission route of TB, suspicious symptoms of TB, negative impact of non-adherence to anti-TB treatment, local TB dispensary, TB free policy (In China, all DS-TB patients could be freely treated with first-line anti-TB drugs at TB dispensaries, and newly diagnosed and suspected TB patients are provided with a free chest X-ray examination and sputum-examination[27]), and curability of TB. Patients who answered ‘Yes’ scored 1, and those who answered ‘No’ scored 0) [28], satisfaction with healthcare, self-reported adherence (SRA) behaviours, and case management and therapy status. In this study, knowledge about TB was defined as patient-centred factors; drug side effects and symptoms were defined as therapy factors; satisfaction with healthcare and received management status were defined as health system factors.

The reliability and validity analysis of the scoring system was conducted to test the validity and consistency of the questionnaire, and the Kaiser–Meyer–Olkin (KMO) and Cronbach’s alpha values were used to evaluate the internal consistency and construct validity of the scoring system [29]. The results indicated that the KMO was 0.826, and Cronbach’s alpha value was 0.719, which indicated that the scoring system had good construct validity and internal consistency (Cronbach’s alpha greater than 0.7, and KMO range from 0.8–0.9) [30].

### Qualitative study

#### Participants

Purposive sampling was used to select DS-TB patients for in-depth interviews and healthcare providers (leaders from the CDC and community health sectors (CHCs), and health care workers (HCWs) from CHCs for key informant interviews. The inclusion and exclusion criteria for patients with TB were consistent with those of the quantitative study. Healthcare providers who are engaged in the management of TB patients were included in this study. The sample size was determined by the number of participants required to reach data saturation.

#### Data collection

Semi-structured topic guides were used in all interviews. The topic guide for participants included adherence behaviours and associated barriers and facilitators from both patients’ and healthcare providers’ perspectives. Three senior researchers conducted interviews in Chinese. Each interview lasted approximately 40–60 min. All interviews were audio-recorded and transcribed for the analysis.

### Data analysis

#### Quantitative analysis

Quantitative data were compiled in Epi Data 3.1, and analysed using Statistical Package for Social Science (SPSS 22.0) (IBM Corporation, Armonk, NY, USA). The test for normality revealed that the numerical data were normal, so numerical variables were reported as mean ± SD and categorical variables as percentages. Missing data were excluded from the analysis. Chi-square (χ^2^) test, T test, and logistic regression analysis were conducted to explore the factors associated with SRA behaviours. A two-tailed probability level of p < 0.05 was selected as the statistically significant level.

#### Qualitative analysis

Each interview was transcribed and reviewed for accuracy. All in-depth interviews were analysed using a framework approach, including familiarizing the data, identifying and coding themes, and summarising and analysing the data [31–33]. Themes that were generated included patient adherence and reasons for non-adherence.

### Definitions

In this study, we defined missed dose, interrupted treatment, and lack of follow-up sputum examination as SRA behaviours.

#### Missed dose

Patients who had forgotten to take their drugs within the last two months [34].

#### Interrupted treatment

Discontinuation of medication for two to eight consecutive weeks before restarting treatment [34].

#### Lack of follow-up sputum exam

Patients did not go to the designated hospital to undergo sputum examination within the prescribed time [34].

## Results

### Characteristic of participants

A total of 465 DS-TB patients were enrolled in the questionnaire survey, and 459 (98.7%) patients were included in this study after quality check of questionnaire. The sociodemographic characteristics are shown in Table 1. Most DS-TB patients were male (70.6%), of Han ethnicity (78.2%), local residents aged 40 or above (79.5%), 82.4% of TB patients lived in rural areas, and 69.7% were married. Patients generally had a low socioeconomic status (83.2%) as defined by the patient education level of junior middle school or below. Almost all patients (96.7%) were covered by basic medical insurance (BMI). Most patients were farmers or migrant workers (68.6%) and had no income (57.7%).

**Table 1:** Univariate analysis between socio-Demographic characteristics and SRA behaviours among DS-TB patients in questionnaire survey (n = 459)

Item	Total	Missed dose	Interrupted treatment	Lack of follow-up sputum exam
	N (%)	N (%)	N (%)	N (%)
Gender				
Male	324 (70.6)	63 (19.4)	29 (9.0)	39 (12.0)
Female	135 (29.4)	24 (17.8)	13 (9.6)	13 (9.6)
Age				
15–19	18 (3.9)	5 (27.8)*	0 (0.0)*	3 (16.7)
20–39	76 (16.6)	23 (30.3)	3 (3.9)	6 (7.9)
40–59	174 (37.9)	27 (15.5)	13 (7.5)	16 (9.2)
≥ 60	191 (41.6)	32 (16.8)	26 (13.6)	27 (14.1)
Ethnicity				
Han	359 (78.2)	58 (16.2)*	34 (9.5)	35 (9.7) ^*^
Others	100 (21.8)	29 (29.0)	8 (8.0)	17 (17.0)
Living region				
Urban area	81 (17.6)	16 (19.8)	12 (14.8)	6 (7.4)
Rural area	378 (82.4)	71 (18.8)	30 (7.9)	46 (12.2)
Residence				
Local residents	437 (95.2)	81 (18.5)	40 (9.2)	49 (11.2)
Migrants	22 (4.8)	6 (27.3)	2 (9.1)	3 (13.6)
Marital status				
Unmarried	69 (15.0)	21 (30.4)*	2 (2.9)	5 (7.2)
Married	320 (69.7)	50 (15.6)	31 (9.7)	38 (11.9)
Divorced/Widowed	70 (15.3)	16 (22.9)	9 (12.9)	9 (12.9)
Education				
Primary and below	257 (56.0)	48 (18.7)	28 (10.9)	32 (12.5)
Junior middle school	125 (27.2)	19 (15.2)	11 (8.8)	14 (11.2)
High school and above	77 (16.8)	20 (26.0)	3 (3.9)	6 (7.8)
Occupation				
Staff/Cadre/Retire	50 (10.9)	6 (12.0)	4 (8.0)	1 (2.0)
Self-employed	10 (2.2)	1 (10.0)	1 (10.0)	1 (10.0)
Farmer/Migrant worker	315 (68.6)	58 (18.4)	27 (8.6)	40 (12.7)
Student	20 (4.4)	5 (25.0)	1 (5.0)	1 (5.0)
Others	64 (13.9)	17 (26.6)	9 (14.1)	9 (14.1)
Health insurance				
Basic medical insurance	444 (96.7)	82 (18.5)	42 (9.5)	52 (11.7)
Non Basic medical insurance	6 (1.3)	3 (50.0)	0 (0.0)	0 (0.0)
No medical insurance	9 (2.0)	2 (22.2)	0 (0.0)	0 (0.0)
Economic sources				
Fixed income	40 (8.7)	4 (10.0)	3 (7.5)	1 (2.5)
No fixed income	154 (33.6)	30 (19.5)	11 (7.1)	19 (12.3)
No income	265 (57.7)	53 (20.0)	28 (10.6)	32 (12.1)

This table presents the results of Univariate analysis between socio-demographic characteristics and SRA behaviours among DS-TB patients, which screening the variables with statistical significant for multivariate analysis

*P < 0.05, SRA refers to Self-reported adherence

Totally 15 participants were included in qualitative study. Two DS-TB patients were included in the in-depth interviews. One male patient was married, a local resident who lived in an urban area, covered by BMI, and had no work or education level of primary school. Another female patient was unmarried; migrants lived in a rural area, covered by BMI, and worked as a clerk in a private enterprise. Both were newly diagnosed treatment patients. A total of 13 healthcare providers, including eight leaders from the CDC, two leaders, and three HCWs from CHCs, were purposively selected for key informant interviews. Leaders from the CDC were in charge of TB prevention and control, and leaders and HCWs from CHCs were in charge of TB patient management.

### Univariate analysis of factors associated with SRA behaviours

**Fig. 1:**
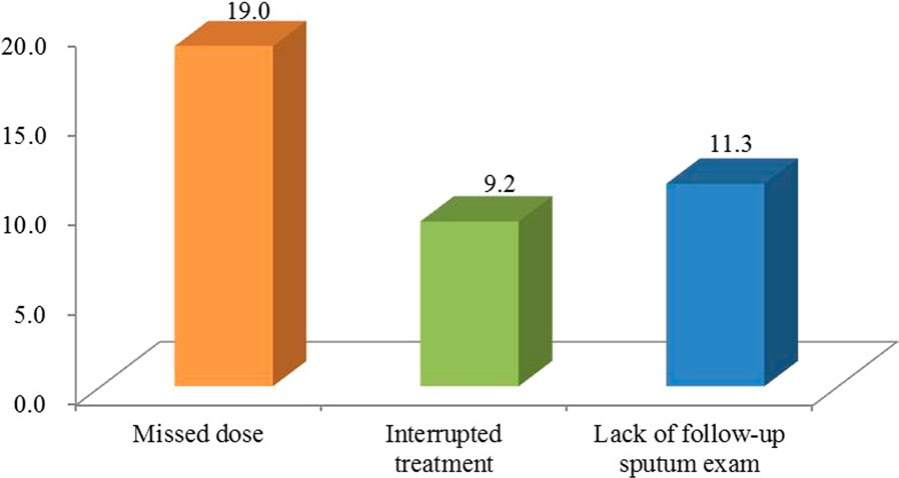
The percentage of non-SRA behaviours among DS-TB patients. This figure presents the non-SRA behaviours among DS-TB patients, including missed dose, interrupted treatment, and lack of follow-up sputum examination

The SRA behaviours are depicted in Fig. 1. Of the DS-TB patients, 19.0% had missed doses higher than interrupted treatment (9.2%) and lacked follow-up sputum examination (11.3%).

#### Socio-demographic factors

As Table 1 demonstrates, patients aged 20–39 and > 60 years had a higher risk of missed dose and interrupted treatment than other age groups (P < 0.05). Unmarried patients had a higher risk of missed doses than the others (P < 0.05). Compared to other ethnicities, Han had a lower risk of missed dose and lack of follow-up sputum examination (P < 0.05).

#### Patient-centred factors

##### Knowledge about TB

As for TB knowledge score, patients who reported interrupted treatment and lack of follow-up sputum exam were scored 2.81 ± 1.50 and 2.87 ± 1.51 respectively, significantly lower than those who adhered to treatment (3.41 ± 1.59) and sputum exam (3.42 ± 1.59) (P < 0.05) (Table 2).

**Table2:** Univariate analysis for the association between patient-centered factors (knowledge scores) and SRA behaviours

SRA behaviors		M ± SD	T	P
Missed dose	Yes	3.21 ± 1.44	0.95	0.343
	No	3.38 ± 1.62		
Interrupted treatment	Yes	2.81 ± 1.50	2.33	0.02^*^
	No	3.41 ± 1.59		
Lack of follow-up sputum exam	Yes	2.87 ± 1.51	2.355	0.019^*^
	No	3.42 ± 1.59		

This table presents the results of Univariate analysis between patient-centered factors (knowledge scores) and SRA behaviours, which screening the variables with statistical significant for multivariate analysis

*P < 0.05, M refers to Mean, SD refers to Standard deviation, SRA refers to Self-reported adherence

#### Family support, therapy, and health system factors associated with SRA behaviours

##### Family support

Most DS-TB patients (94.6%, n = 434) received family support, including psychological (66.2%, n = 304), economic support (20.0%, n = 92), and nutritional support (8.3%, n = 38). As Table 3 demonstrates, family support was significantly associated with missed dose (P < 0.05), but no difference was observed between interrupted treatment, lack of follow-up examination, and family support (P > 0.05).

**Table 3:** Univariate analysis of family support, therapy and health system factors associated with SRA behaviours

Items	Total	Missed dose	Interrupted treatment	Lack of follow-up sputum exam
	N (%)	N (%)	N (%)	N (%)
Family support factors				
Economic	92 (20.0)	24 (26.1%)*	9 (9.8%)	7 (7.6%)
Nutritional	38 (8.3)	4 (10.5%)	6 (15.8%)	7 (18.4%)
Psychological	304 (66.2)	51 (16.8%)	26 (8.6%)	34 (11.2%)
No	25 (5.4)	8 (32.0%)	1 (4.0%)	4 (16.0%)
Therapy factors				
Drug side- effect				
Yes	249 (54.2)	46 (18.5)	31 (12.4)*	34 (13.7)
No	210 (45.8)	41 (19.5)	11 (5.2)	18 (8.6)
Symptoms				
Yes	406 (88.5)	77 (19.0)	35 (8.6)	44 (10.8)
No	53 (11.5)	10 (18.9)	7 (13.2)	8 (15.1)
Health system factors				
Satisfied with management from HCWs in CDC/TB dispensary				
Yes	424 (92.4)	80 (18.9)	32 (7.5)*	45 (10.6)
No	32 (7.0)	7 (21.9)	9 (28.1)	6 (18.8)
No management	3 (0.7)	0 (0.0)	1 (33.3)	1 (33.3)
Satisfied with management from HCWs in CHCs				
Yes	417 (90.8)	76 (18.2)	37 (8.9)	44 (10.6)
No	8 (1.7)	2 (25.0)	2 (25.0)	1 (12.5)
No management	34 (7.4)	9 (26.5)	3 (8.8)	7 (20.6)
Satisfied with management from HCWs in village clinics				
Yes	240 (52.3)	34 (14.2) ^*^	24 (10.0)	27 (11.3)
No	4 (0.9)	3 (75.0)	0 (0.0)	1 (25.0)
No management	215 (46.8)	50 (23.3)	18 (8.4)	24 (11.2)

This table presents the results of Univariate analysis of family support, therapy and health system factors associated with SRA behaviours, which screening the variables with statistical significant for multivariate analysis

*P < 0.05, SRA refers to Self-reported adherence, HCWs refers to Healthcare Workers, CHCs refers to Community Health Sectors, CDC refers to Centers for Disease Control and Prevention, TB refers to Tuberculosis

##### Therapy factors

Of the patients with DS-TB, 54.2% reported drug side-effects, and most (88.5%) patients had symptoms (Table 3). Compared with patients who reported no drug side effects, patients who reported drug side-effects had a higher risk of interrupted treatment (P < 0.05). No significant association was observed between the symptoms and SRA behaviours (P > 0.05).

##### Health system factors

In total, 92.4%, 90.8%, and 52.3% of DS-TB patients were satisfied with the management of HCWs in CDC/TB dispensaries, CHCs, and village clinics, respectively. Furthermore, 0.7%, 7.4%, and 46.8% of DS-TB patients self-reported that they did not receive management from HCWs in CDC/TB dispensaries, CHCs, and village clinics, respectively. Patients who were satisfied with the management of HCWs in the CDC/TB dispensary had a lower risk of interrupted treatment when compared with patients who self-reported not being satisfied (7.5% versus 28.1%) and did not receive the management (7.5% versus 33.3%) from HCWs in the CDC/TB dispensary (P < 0.05). Patients who were satisfied with the management of HCWs in village clinics had a lower risk of missed dose when compared with patients who self-reported not being satisfied (14.2% versus 75.0%) and not receiving HCWs in village clinics (14.2% versus. 23.3%) (P < 0.05) (Table 3). No association was observed between satisfaction with the management of HCWs in CHCs and SRA behaviours (P > 0.05).

### Multivariate analysis of factors associated with SRA behaviours

Results of binary logistic regression analysis (Table 4) demonstrated that DS-TB patients aged 20–39 had a higher risk of missed dose than patients who were older than 60 years [OR (95% CI): 2.302 (1.001–5.305)], Han ethnicity [OR (95% CI): 0.524 (0.301–0.912)], received psychological support [OR (95% CI): 0.379 (0.144–0.998)] from their family when they received TB treatment had a lower risk of missed dose than other ethnicities and patients who received no support, respectively. Patients aged 20–39 had a lower risk of interrupted treatment than patients aged > 60 years (OR [95% CI]: 0.278 (0.077–0.982)], patients who had drug side-effects had a higher risk of interrupted treatment than patients who had no drug side-effects [OR (95% CI): 2.587 (1.237–5.412)]. Notably, patients who scored higher knowledge had a lower risk of lack of follow-up sputum examination (OR [95% CI]: 0.817 [0.673–0.991]).

**Table 4:** Multivariate analysis of Factors associated with SRA behaviours

Items	Missed dose	Interrupted treatment	Lack of follow-up sputum exam
	OR (95%CI)	OR (95%CI)	OR (95%CI)
1. Socio-demographic factors			
Age			
≥ 60	Reference	Reference	–
40–59	0.967 (0.532–1.756)	0.596 (0.285–1.245)	–
20–39	2.302 (1.001–5.305)*	0.278 (0.077–0.982) ^*^	–
15–19	1.915 (0.473–7.750)	–	–
Ethnicity			
Others	Reference	–	Reference
Han	0.524 (0.301–0.912)*	–	0.590 (0.312–1.117)
Marital status			
Divorced/Widowed	Reference	–	–
Unmarried	1.015 (0.352–2.927)	–	–
Married	0.726 (0.363–1.452)	–	–
2. Patient-centered factors			
Knowledge score	*–*	0.886 (0.708–1.109)	0.817 (0.673–0.991)*
3. Family support factors			
No support	Reference	–	–
Economic support	0.659 (0.233–1.867)	–	–
Nutritional support	0.249 (0.061–1.020)	–	–
Psychological support	0.379 (0.144–0.998)*	–	–
4. Therapy factors			
Drug side-effect			
No	–	Reference	–
Yes	–	2.587 (1.237–5.412)*	–
5. Health system factors			
Satisfied with management from doctors in CDC/TB dispensary			
No management	–	Reference	–
Yes	–	0.160 (0.011–2.428)	–
No	–	0.632 (0.038–10.302)	–
Satisfied with management from HCWs in village clinics			
No management	Reference	–	–
Yes	0.623 (0.374–1.040)	–	–
No	9.700 (0.914–102.906)	–	–

This table presents the results of Multivariate analysis of Factors associated with SRA behaviours, including socio-demographic characteristics, patient-centered factors, family support, therapy and health system factors

OR refers to odds ratio, CI refers to confidence interval, SRA refers to Self-reported adherence, HCWs refers to Healthcare Workers, CDC refers to Centers for Disease Control and Prevention, TB refers to Tuberculosis

*P < 0.05, “–”refers to this variable was not included in the logistic model for this independent variable

### Qualitative results about reasons for non-SRA behaviours

The results of the qualitative study are shown in Table 5. Key informant interviews indicated that inadequate knowledge about TB and financial difficulties were the main reasons for non-SRA behaviours in DS-TB patients. Interviews from both patients and HCWs reported that most patients were satisfied with the management of HCWs, and few patients were afraid that there were some problems with drug intake.

**Table 5:** Qualitative results of reasons for non-SRA behaviours

Themes	Results	Quotation
Health literacy of patients	Patients with low health literacy, lack of adequate knowledge about TB treatment had higher risk of non-adherence behaviors	"There is one patient had few knowledge about TB and he stopped take drugs after took 2 times drug for he feels better…" (Leader from CDC) "Patients didn't believe standard regimens and the prefer to choose folk remedies to therapy TB…" (HCWs from CHCs)
Financial burden	Heavy financial burden was one of the reasons for poor adherence behaviors	"Patients from rural region were difficulty in take drugs regularly, and most of the patients had financial difficulty and they can't afford the charge of examination" (Leader from CDC) "Patients who didn't adherence to treatment for following reasons: their family was difficulty; they are afraid of troublesome and too many examination" (Leaders from CHCs)
Health system management	Most patients were satisfied with the management of HCWs but few patients had less willingness for drug intake	"The doctor from our village hospital, he always come to our home and we live close to each other…" (Male patient) "I am satisfied with the management from HCWs in CHCs during the treatment period….." (Female patient) "Most patients satisfied with our management, especially the patients from countryside, they are happy for receiving our management…." (Leaders from CDC) "…Every time we came to the house of patients they will say lot of words to appreciate our work " (Leaders from CHCs) "One aged patient always say thanks to us for our management, but he didn’t want take drug for some inconvenient reasons" (HCWs from CHCs)

This table presents the qualitative results of reasons for non-SRA behaviours, which indicated the health literacy of patients, financial burden and health system management were the reasons of non-SRA behaviours

SRA refers to Self-reported adherence, HCWs refers to Healthcare Workers, CHCs refers to Community Health Sectors, CDC refers to Centers for Disease Control and Prevention, TB refers to Tuberculosis

## Discussion

Standard treatment regimens in DOTs require six months of treatment for initial TB patients and eight months for retreatment TB patients [9]. Adherence to long-term treatment was the biggest challenge for TB control, and poor adherence behaviours were common during TB treatment despite various interventions being implemented to improve it [11]. Currently, the main obstacle for improving adherence behaviours is the lack of understanding of the barriers and facilitators of adherence to TB treatment. Therefore, this study identified the factors associated with SRA behaviours in DS-TB patients, including socio-demographic, patient-centred, family support, health system, and therapy factors, to provide evidence-based strategies to improve SRA behaviours.

In this study, SRA behaviours included missed dose, interrupted treatment, and lack of follow-up sputum examination. We found that almost 20% of DS-TB patients had missed doses, which occurred most frequently, followed by a lack of follow-up sputum examination and interrupted treatment. The incidence of poor adherence was much higher in a previous study on MDR-TB patients in Chongqing [34] but lower than that in DS-TB patients reported in a previous study [25].

Unlike the previous study, patients with MDR-TB were mainly young and middle-aged [35]. Most DS-TB patients in this study were aged more than 60 years, which was also inconsistent with our previous study in which most DS-TB patients were young and middle-aged [25]. This result may indicate that older people are increasingly vulnerable to TB development in Southwest China. Age has been shown to be an important factor associated with treatment adherence [36], which was mainly attributed to the age distribution of TB. However, this study found that most DS-TB patients were aged, but they had a lower risk of missed dose and higher risk of interrupted treatment than patients aged 20–39. We supposed that younger patients might be busy at work and have lighter symptoms, so they were more likely to forget to take medication, but they were still committed to completing the treatment. This study also found that Han ethnicity had better SRA behaviours, which is consistent with previous studies [35, 37]. Southwest China consists of many minorities, especially the Guizhou and Yunan provinces [38], and most of the minorities had a lower socioeconomic status, culture, and education compared with Han ethnicity [35], which might be associated with poor adherence. Studies have reported that economic constraints may reduce patients’ ability to adhere to TB treatment [39, 40]. The qualitative results of this study also revealed that financial burden was the main reason for non-SRA behaviours. In addition, a previous study found that patients with low education had a higher risk of non-SRA behaviours [41–44], which was not observed in this study.

Inadequate knowledge about TB was a determinant factor for adherence behaviours, which has been reported in many previous studies [45–48]. Our results from both quantitative and qualitative research consistently found that patients who scored higher on TB knowledge had a lower risk of lack of follow-up sputum examination. Therefore, health education on TB knowledge should be intensified among TB patients to improve their health literacy in the future.

Previous studies indicated that patients who received social support from friends and families were more likely to take their medication regularly; besides, financial and food support were the main factors associated with adherence [45, 46, 49, 50], and family support was a protective factor for DOTs [45]. This study also found that psychological support was the main factor associated with SRA behaviours. Patients who received psychological support from their families exhibited better SRA behaviours. Studies have reported that psychological support from reliable members, including family or friends, could re-establish belief in the curability of TB [46, 49]. Many patients worried about the curability of TB, had a stigma, and were exposed to discrimination from others [51–53]. Hence, it is crucial for family members to support and encourage patients to acknowledge TB, and to further help patients adhere to TB treatment.

Previous studies reported that drug side-effects were the major reasons for poor adherence behaviours [45, 48, 52, 54], which was consistent with our study that reported that patients who had drug side-effects had a higher risk of interrupted treatment. Pill burden was thought to be a factor for non-adherence to TB medication among patients with TB [44]. However, this study indicated that there was no association between missed dose, lack of follow-up sputum examination, and drug side-effects, which might be attributed to most DS-TB patients aged more than 60 years with a higher risk of interrupted treatment and a lower risk of missed dose and lack of follow-up sputum examination in our study. A study from Hubei, China reported that patients aged > 60 years had a higher risk of drug side effects than patients aged < 60 years [55]. Therefore, more attention should be paid to the drug side effects of TB medicine among aged TB patients in order to improve their adherence to treatment.

Many studies have indicated that the health system is a major factor influencing adherence behaviours for TB patients, including case management from CHCs and satisfaction with healthcare services [51, 53, 56, 57]. This study investigated the satisfaction of TB patients with case management from HCWs in CDC/TB dispensaries, CHCs, and village clinics. Unfortunately, no association was observed between health system factors and SRA behaviours, which may be attributed to the lack of effective indicators to evaluate the health system factors in this study. The quality of the healthcare system should be evaluated in future studies.

## Limitations

This study had several limitations. First, only two of the 88 counties in Guizhou province were selected as representative counties of Guizhou, and the sample counties would increase in future studies to ensure good representation. Second, information bias existed in this study for some patients with low education levels, and they had a poor understanding of the contents of the questionnaire.

## Conclusions

Poor adherence to treatment is one of the biggest challenges for TB control. Strategies to improve health literacy and strengthen psychological support are urgently needed for TB patients. More attention should be paid to patients from minorities with drug side-effects, aged 20–39 and over 60 years old. Patient-centred intervention measures should be designed and implemented to improve adherence behaviours.

## Data Availability

The datasets generated and/or analyzed during the current study are available from the corresponding author on reasonable request.

## Abbreviations

DS-TB: Drug-sensitive tuberculosis
SRA: Self-reported adherence
CHCs: Community Health Sectors
HCWs: Healthcare workers
CDC: Centers for Disease Control and Prevention
TB: Tuberculosis
WHO: World Health Organization
MDR-TB: Multidrug-resistant TB
RFP: Rifampicin
RR-TB: Rifampicin-resistant TB
DOTs: Direct Observation of Treatment, short course
KMO: Kaiser–Meyer–Olkin

## References

[1] World Health Organization (2020). Global tuberculosis report, 2020.

[2] Orcau À, Caylà JA, Martínez JA (2011). Present epidemiology of tuberculosis. Prevention and control programs. Enferm Infecc Microbiol Clin.

[3] Pearce L (2017). Tuberculosis. Emerg Nurse.

[4] The Fifth National Tuberculosis Epidemiological Sampling Survey Technical Steering Group (2012). The fifth national tuberculosis epidemiology sampling survey report. Chin J Ant tuberculosis.

[5] Alipanah N, Jarlsberg L, Miller C, Linh NN, Falzon D, Jaramillo E (2018). Adherence interventions and outcomes of tuberculosis treatment: A systematic review and meta-analysis of trials and observational studies. PLoS Med.

[6] Wang Y, Chen H, Huang Z, McNeil EB, Lu X, Chongsuvivatwong V (2019). Drug non-adherence and reasons among multidrug-resistant tuberculosis patients in Guizhou, China: a cross-sectional study. Patient Prefer Adherence.

[7] Nezenega ZS, Perimal-Lewis L, Maeder AJ (2020). Factors influencing patient adherence to tuberculosis treatment in Ethiopia: a literature review. Int J Environ Res Public Health.

[8] World Health Organization (2003). Adherence to long-term therapies evidence for action.

[9] World Health Organization (2002). An expanded DOTS framework for effffective tuberculosis control. Int J Tuberc Lung Dis.

[10] World Health Organization. Companion Handbook to the WHO Guidelines for the Programmatic Management of Drug-resistant Tuberculosis. Geneva: WHO/HTM/TB/2014.11. Geneva, Switzerland: WHO; 2014. 25320836

[11] Munro SA, Lewin SA, Smith HJ, Engel ME, Fretheim A, Volmink J (2007). Patient adherence to tuberculosis treatment: a systematic review of qualitative research. PLoS Med.

[12] Loveday M, Wallengren K, Brust J (2015). Community-based care vs. centralized hospitalization for MDR-TB patients, KwaZulu-Natal, South Africa. Int J Tuberc Lung Dis.

[13] Wai PP, Shewade HD, Kyaw NTT (2018). Community based MDR-TB care project improves treatment initiation in patients diagnosed with MDR-TB in Myanmar. PLoS ONE.

[14] Jaiswal A, Singh V, Ogden JA, Porter JDH, Sharma PP (2003). Adherence to tuberculosis treatment: lessons from the urban setting of Delhi, India. Trop Med Int Health.

[15] Singh V, Jaiswal A, Porter JDH, Ogden JA, Sarin R (2002). TB control, poverty, and vulnerability in Delhi, India. Trop Med Int Health.

[16] Greene JA (2004). An ethnography of non-adherence: Culture, poverty, and tuberculosis in urban Bolivia. Cult Med Psychiatry.

[17] Nájera-Ortiz JC, Sánchez-Pérez HJ, Ochoa-Díaz H, Arana-Cedeño M, Lezama MS, Mateo MM (2008). Demographic, health services and socio-economic factors associated with pulmonary tuberculosis mortality in Los Altos Region of Chiapas, Mexico. Int J Epidemiol.

[18] Okeyo ILA, Dowse R (2018). An illustrated booklet for reinforcing community health worker knowledge of tuberculosis and facilitating patient counselling. Afr J Prim Health Care Fam Med.

[19] Lin HR. The effects and influence factors of DOT strategy in pulmonary tuberculosis[D]. Qingdao University, 2016 (**in Chinese**).

[20] Hou WL, Song FJ, Zhang NX, Dong XX, Cao SY, Yin XX (2012). Implementation and community involvement in DOTS strategy: a systematic review of studies in China. Int J Tuberc Lung Dis.

[21] Hu DY, Liu XY, Chen J, Wang Y, Wang T, Zeng W (2008). Direct observation and adherence to tuberculosis treatment in Chongqing, China: a descriptive study. Health Policy Plan.

[22] Zou GY, Wei XL, Walley JD, Yin J, Sun Q (2012). Factors influencing integration of TB services in general hospitals in two regions of China: a qualitative study. BMC Health Serv Res.

[23] Zhao Y, Cui S, Yang J, Wang W, Guo A, Liu Y (2011). Basic public health services delivered in an urban community: a qualitative study. Public Health.

[24] Chen W, Xia YY, Li T, Chen H (2016). Analysis for the Global and China TB Epidemic Situation in 2015. J Tuber Lung Health.

[25] Pu J, Chen W, Jiang WX (2019). Is tuberculosis patients management improved in the integrated TB control model in West China? A survey in Guizhou Province, China. Infect Dis Poverty.

[26] Vermeire E, Hearnshaw H, van Royen P, Denekens J (2001). Patient adherence to treatment: three decades of research. A comprehensive review. J Clin Pharmacol Ther.

[27] Jia X, Chen J, Zhang S, Dai B, Long Q, Tang S (2016). Implementing a "free" tuberculosis (TB) care policy under the integrated model in Jiangsu, China: practices and costs in the real world. Infect Dis Poverty.

[28] General Office of the National Health and Family Planning Commission, PRC. Key information and Knowledge points of Tuberculosis prevention and Treatment. National Health and Family Planning Commission; April 2016 (**in Chinese**).

[29] George D, Mallery P (1999). SPSS for windows step by step: a simple guide and reference.

[30] Nunnally J, Bernstein I (1994). Psychometric theory.

[31] Smith J, Firth J (2011). Qualitative data analysis: the framework approach. Nurse Research.

[32] Srivastava A, Thomson SB (2009). Framework analysis: a qualitative methodology for applied policy research. J Adm Gov.

[33] Ward DJ, Furber C, Tierney S, Swallow V (2013). Using framework analysis in nursing research: a wirked example. J Adv Nurs.

[34] Xing W, Zhang R, Jiang W (2021). Adherence to multidrug resistant tuberculosis treatment and case management in chongqing, china - a mixed method research study. Infect Drug Resist.

[35] Zhou QB. Study on epidemic characteristics and the risk factors of anti-tuberculosis treatment effect of multidrug-resistance tuberculosis in Yunan province[D]. Kunming Medical University, 2017 (**in Chinese**).

[36] Krasniqi S, Jakupi A, Daci A (2017). Tuberculosis Treatment Adherence of Patients in Kosovo. Tuberc Res Treat.

[37] Zhen T. The application research of extended service of nursing based on the network platform for patients with tuberculosis of Xinjiang region[D]. Xinjiang Medical University, 2017 (**in Chinese**).

[38] Chen T. The effect and evaluation for interventions to vulnerable people of TB control project in China[D]. Chongqing Medical University, 2011 (**in Chinese**).

[39] Diriba Daksa M, Melaku Kebede T, Dahjejot M (2016). Patients’ adherence to anti-tuberculosis medicines and associated factors for non-adherence at a tertiary teaching hospital South West Ethiopia. Eur J Ther.

[40] Daba M, Tesfaye M, Adorjan K, Krahl W, Tesfaye E, Yitayih Y, Strobl R, Grill E (2019). Khat and alcohol use disorders predict poorer adherence to anti-tuberculosis medications in southwest ethiopia: a prospective cohort study. Prepr Lancet..

[41] Yasin Mohammed A, Kaso Adem M (2014). Treatment adherence among tuberculosis and human immuno defificiency virus coinfected patients in ginnir referral hospital. Am J Public Health Res.

[42] Tola HH, Garmaroudi G, Shojaeizadeh D (2017). The effect of psychosocial factors and patients’ perception of tuberculosis treatment non-adherence in Addis Ababa, Ethiopia. Ethiop J Health Sci.

[43] Tadesse T, Demissie M, Berhane Y, Kebede Y, Abebe M (2013). Long distance travelling and financial burdens discourage tuberculosis DOTs treatment initiation and compliance in Ethiopia: a qualitative study. BMC Public Health.

[44] Gebremariam MK, Bjune GA, Frich JC (2010). Barriers and facilitators of adherence to TB treatment in patients on concomitant TB and HIV treatment: a qualitative study. BMC Public Health.

[45] Tekle B, Mariam DH, Ali A (2002). Defaulting from DOTS and its determinants in three districts of Arsi Zone in Ethiopia. Int J Tuberc Lung Dis.

[46] Michael KW, Belachew T, Jira C (2004). Tuberculosis defaulters from the “dots” regimen in Jimma zone, southwest Ethiopia. Ethiop Med J.

[47] Woimo TT, Yimer WK, Bati T, Gesesew HA (2017). The prevalence and factors associated for anti-tuberculosis treatment non-adherence among pulmonary tuberculosis patients in public health care facilities in South Ethiopia: a cross-sectional study. BMC Public Health.

[48] Kiros YK, Teklu T, Desalegn F, Tesfay M, Klinkenberg E, Mulugeta A (2014). Adherence to anti-tuberculosis treatment in Tigray Northern Ethiopia. Public Health Action.

[49] Mindachew M, Deribew A, Memiah P, Biadgilign S (2014). Perceived barriers to the implementation of Isoniazid preventive therapy for people living with HIV in resource constrained settings: a qualitative study. Pan Afr Med J.

[50] Eticha T, Kassa E (2014). Non-adherence to anti-TB drugs and its predictors among TB/HIV co-infected patients in Mekelle Ethiopia. J Bioanal Biomed.

[51] Gugssa Boru C, Shimels T, Bilal AI (2017). Factors contributing to non-adherence with treatment among TB patients in Sodo Woreda, Gurage Zone, Southern Ethiopia: a qualitative study. J Infect Public Health.

[52] Ayele HT, van Mourik MS, Bonten MJ (2016). Predictors of adherence to isoniazid preventive therapy in people living with HIV in Ethiopia. Int J Tuberc Lung Dis.

[53] Mesfifin MM, Newell JN, Walley JD, Gessessew A, Tesfaye T, Lemma F, Madeley RJ (2009). Quality of tuberculosis care and its association with patient adherence to treatment in eight Ethiopian districts. Health Policy Plan.

[54] Tesfahuneygn G, Medhin G, Legesse M (2015). Adherence to Anti-tuberculosis treatment and treatment outcomes among tuberculosis patients in Alamata District, northeast Ethiopia. BMC Res Notes.

[55] Sun XG, Sun F (2007). Analysis of side effects of anti-tuberculosis drugs in 574 patients with smear positive tuberculosis. Guide China Med..

[56] Nezenega ZS, Gacho YH, Tafere TE (2013). Patient satisfaction on tuberculosis treatment service and adherence to treatment in public health facilities of Sidama zone South Ethiopia. BMC Health Serv Res.

[57] Mekonnen HS, Azagew AW (2018). Non-adherence to anti-tuberculosis treatment, reasons and associated factors among TB patients attending at Gondar town health centers Northwest Ethiopia. BMC Res Notes.

